# Longitudinal anthropometry of children and adolescents using 3D-body scanning

**DOI:** 10.1371/journal.pone.0203628

**Published:** 2018-09-13

**Authors:** Henry Loeffler-Wirth, Mandy Vogel, Toralf Kirsten, Fabian Glock, Tanja Poulain, Antje Körner, Markus Loeffler, Wieland Kiess, Hans Binder

**Affiliations:** 1 Interdisciplinary Centre for Bioinformatics, Leipzig University, Leipzig, Germany; 2 LIFE, Leipzig Research Center for Civilization Diseases; Leipzig University, Leipzig, Germany; 3 Hospital for Children and Adolescents, Centre for Pediatric Research; Leipzig University, Leipzig, Germany; 4 Institute for Medical Informatics, Statistics and Epidemiology, Leipzig University, Leipzig, Germany; Vanderbilt University, UNITED STATES

## Abstract

3D-body scanning anthropometry is a suitable method for characterization of physiological development of children and adolescents, and for understanding onset and progression of disorders like overweight and obesity. Here we present a novel body typing approach to describe and to interpret longitudinal 3D-body scanning data of more than 800 children and adolescents measured in up to four follow-ups in intervals of 1 year, referring to an age range between 6 and 18 years. We analyzed transitions between body types assigned to lower-, normal- and overweight participants upon development of children and adolescents. We found a virtually parallel development of the body types with only a few transitions between them. Body types of children and adolescents tend to conserve their weight category. 3D body scanning anthropometry in combination with body typing constitutes a novel option to investigate onset and progression of obesity in children.

## Introduction

Anthropometry is important for understanding the development of children and adolescents. It allows for detailed evaluation of diversity of body shapes and their variations in the context of aging and disorders like overweight, obesity and their comorbidities [1]. Epidemiologists increasingly use a life course approach with special interest for early patterns of growth because of their association with diseases in later life, such as obesity, type 2 diabetes, hypertension or stroke [2]. For example, overweight children showed a more than twofold risk of becoming overweight adults compared to normal weight children. Life course studies often comprise two steps where the first step consists of describing and modeling the growth data, and the second step aims at studying associations of the model with determinants of disorders. Several parametrical growth models have been developed to describe singular measures such as infants weight or height as a function of age either for single individuals or for mean measures averaged over populations (see [2] and references cited therein). However, these parametric models are less suited to describe the diversity of growth pattern in a population and, moreover, they are prone to overfitting.

An alternative approach uses developmental trajectory-types to assess the relation between different body shapes in early/middle life and the mortality risk [3]. This study of Song et al. classified participants into five distinct, mutually exclusive trajectory groups. It allows for detailed evaluation of the population heterogeneity in terms of body shapes called ‘somatotypes’ over the life course, and it permits direct comparison of the mortality risk across these groups. It was found that heavy body shape in early and middle life, especially when accompanied by weight increase in middle life, was associated with higher mortality. In contrast, people who maintained a stably lean body shape had the lowest mortality. These results indicate health benefit of body shape management across the lifespan.

In the last years, digital laser-scanning anthropometry has replaced calipers, weight scales, tape measures and other tools that have been used for centuries to assess body dimensions [4]. Three-dimensional (3D-) whole body scanning provides a promising technique to gather anthropometric data, granting opportunity to assess dozens of individual body measures at once with high accuracy and within only a few seconds of time [5]. This technological transformation is so rapid and profound that the field has yet to accommodate by development of new concepts. Particularly, the capability of 3D laser-scanning bodymetry arises from the vast number of measured body surface dimensions that allow for the discovery of detailed health risk phenotypes far beyond the popular “apple-pear” classification.

3D-body scanning was systematically applied in the Leipzig Research Center for Civilization Diseases (LIFE). The LIFE Child study is one of the largest longitudinal studies with an extensive phenotyping of urban children and adolescents in Germany [6,7]. Based on the data collected, we recently developed new shape indices called body types, which have the potential to predict health outcomes [8–10]. These body types associate with specific weight and age ranges (see Table 1), where ‘younger’, `medium age’ and `older’ body types emerge and outgrow in successive order. The cross-sectional data on 2,735 children and adolescents are now complemented by longitudinal data of 808 participants in the age range between 6 and 18 years. For this, participants were assessed up to 4 times in intervals of 1 year.

**Table 1:** Age and BMI ranges of the body types in the longitudinal data.

Body type	Age (y) ^1^	BMI ^1^
Young age, normal weight	9.1 ± 1.6	16.4 ± 1.9
Medium age, lower weight	12.7 ± 2.0	17.3 ± 2.0
Medium age, normal weight	11.7 ± 1.6	18.4 ± 2.3
Medium age, overweight / obese	10.8 ± 2.2	24.2 ± 5.4
Older age, normal weight	14.6 ± 1.5	20.6 ± 2.1
Older age, high weight	14.9 ± 1.6	22.9 ± 2.7
Older age, overweight / obese	13.4 ± 1.9	26.4 ± 4.7

1: average value ± standard deviation

Here we present these novel longitudinal data to address the question how the body types change during child development. Particularly we ask about age-dependent transitions between the different body types, an information which is absent in the cross sectional data, and whether children and adolescents tend to switch between weight categories (underweight, normal and overweight) upon development or tend to maintain their category.

## Material and methods

The LIFE Child study has been registered with the trial number NCT02550236 and was approved by the Ethics Committee of the University of Leipzig (Reg. No. 264-10-19042010). As a prerequisite to enrolment, written informed consent was obtained from all participants or their parents. All procedures performed in studies involving human participants were in accordance with the ethical standards of the institutional research committee and with the 1964 Helsinki declaration and its later amendments.

We utilized 3D-body scanning data, which were generated in the frame of LIFE Child using a ‘Vitus Smart XXL’ laser scanner in combination with AnthroScan 2.9.9 software, both complying with the ISO 20685 international standard. This data was then preprocessed and clustered into body types as described in [11]. The study presented here comprises longitudinal data on 808 children and adolescent participants measured in one to four follow-ups in one-year intervals. We stratified this data according to age at measurement and corresponding body type. Then transitions between age intervals and/or body types were counted for all follow-up measurements.

## Results

Our previous analysis of cross sectional body scanner data of 2,735 children and adolescents of age 6 to 18 years provided seven body types [11]. These body types are sex-invariant and differentiate the participants’ body shapes in terms of three weight (lower, normal, and high/overweight) and three age (young, medium and older) categories. For younger children (age of 5–10 years) we found a common ‘early childhood body shape’ which later splits into three weight-dependent types for older children, with a one to two years delay for boys.

**Fig 1:**
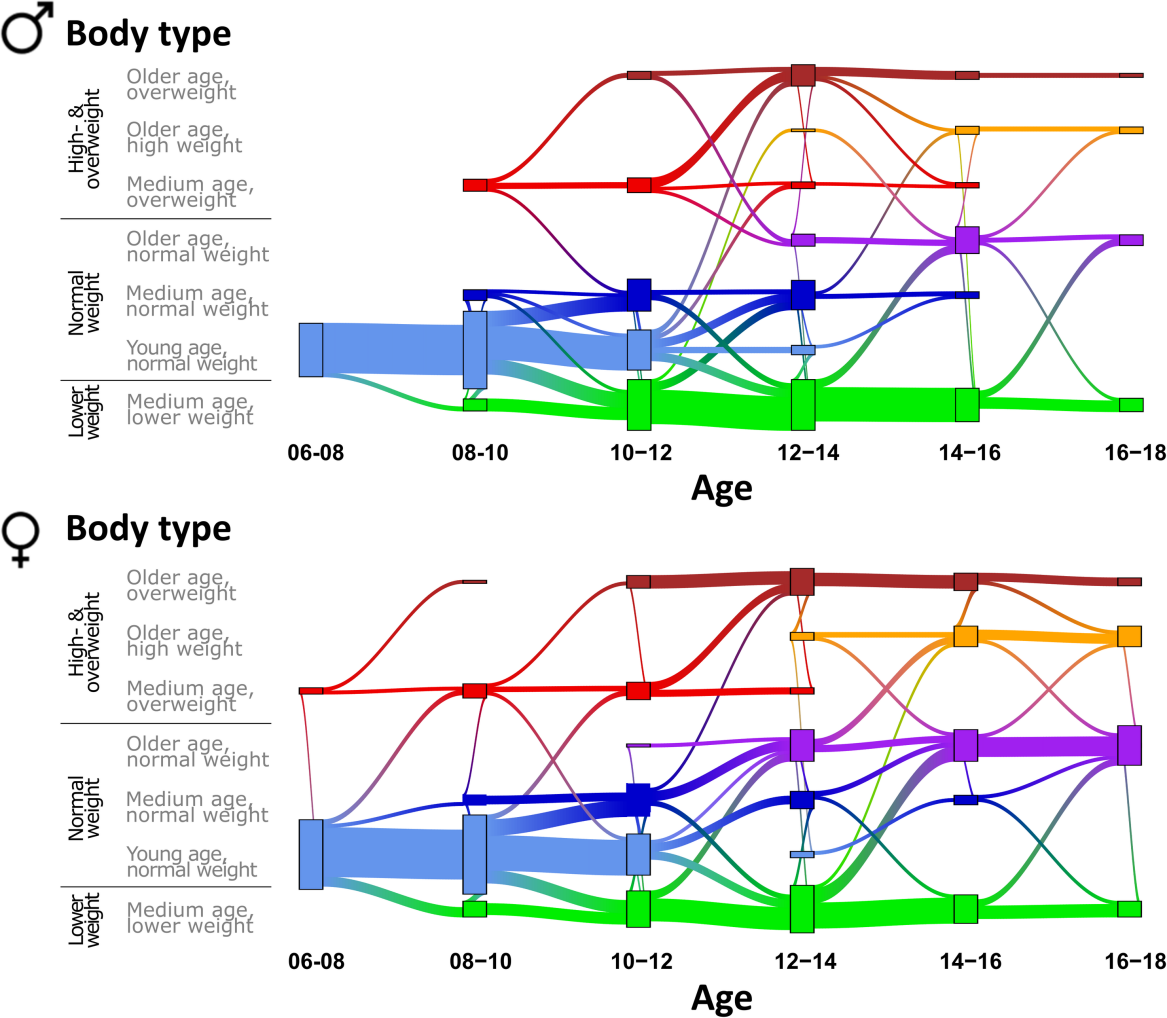
Body type trajectories upon development. Longitudinal assignment of body types and of the transitions between them in follow-up measurements shows a trend from ‘younger’ to ‘older’ body types, and the conservation of the weight category. The connections indicate transitions between the body types obtained from the measurements of the same participants.

Here we analyzed longitudinal 3D-body scanning data of 808 children and adolescent taken from the original cohort to assess aspects of developing body types. After assigning each measurement of a participant to one of the seven body types and stratification into two-years age-intervals, we counted the transitions between age intervals and/or body types, and visualized these transitions in terms of a flow diagram (Fig 1). The vertical dimension in Fig 1 describes changes of body type assignment in a follow-up measurement, whereas the horizontal dimension represents age at measurement stratified by intervals of two-years.

Up to an age of about 10 years, most of the children were assigned to the ‘young age/normal weight’ body type. Then, this young age/normal weight-type starts to split into medium age (low weight and medium weight) types. However, about 50% still remain in the young age-type up to an age of 12 years before they switch into medium age types in the next two years of development. Hence, participants with ‘young age / normal weight’ body type are predominantly assigned to ‘medium age / normal weight’ and, to a less degree, ‘medium age / lower weight’ body types in follow-up measurements. Overall, we find a general shift from ‘younger’ to ‘older’ body types during development as expected. Interestingly, the ‘medium age / lower weight’ body type also comprises some older participants up to an age of 18 years, whose lean body shape is similar to that of younger participants.

Importantly, these trajectories narrow down to essentially three horizontal paths associated with high- and overweight, normal weight, and lower weight categories, respectively (see assignment of body types in Fig 1). This applies to both girls and boys. However, for girls this trend starts one to two years earlier than for boys in agreement with the cross-sectional data [11]. In total, about 70% of participants remain in the same category in all follow-up assessments, 52% even conserve the body type throughout the study. Remarkably, only about 3% of the transitions switch into the overweight category, e.g. grow from normal-weight and lower-weight body types into overweight ones. Vice versa, only 2% of the transitions switch from overweight into normal weight category, hinting that signs of later overweight and obesity incidence are already observable in the age range under study.

For older participants, transitions to heavier weight body types can be observed more frequently than transitions to lower weight body types.

## Discussion and conclusion

Body type trajectories from longitudinal measurements indicate an almost parallel development of lower-, normal- and overweight children and adolescents, who mainly maintain their weight category with only a relatively small number of transitions. The category of high- and overweight collects about 15% of measurements of boys and 20% of girls, potentially indicating increased risk for a series of civilization diseases such as diabetes and cancer as reported in two prospective studies in the USA [3]. Meta-studies and the WHO standards demonstrate that healthy children, raised in good environmental conditions and following recommended feeding practices, have strikingly similar patterns of growth independent of ancestry or residence [12].

Our results however need further investigation: Firstly, the covered age range should be extended towards adults to study changes into overweight categories at later ages, e.g. an instantaneous trend towards increased weight during life span previously reported [3]. Secondly, the body types should be associated with the long-term health status of the participants to estimate their potential risk for selected diseases.

This life course perspective is crucial for a better understanding of the health consequences of overweight and obesity, and for development of effective prevention strategies. Hereby, anthropometrical characterization of developing and aging populations in terms of body types and of transitions between them constitutes a novel option to investigate onset and progression of obesity and other civilization diseases.
